# Relaxin Prevents Cardiac Fibroblast-Myofibroblast Transition via Notch-1-Mediated Inhibition of TGF-β/Smad3 Signaling

**DOI:** 10.1371/journal.pone.0063896

**Published:** 2013-05-21

**Authors:** Chiara Sassoli, Flaminia Chellini, Alessandro Pini, Alessia Tani, Silvia Nistri, Daniele Nosi, Sandra Zecchi-Orlandini, Daniele Bani, Lucia Formigli

**Affiliations:** Department of Experimental and Clinical Medicine - Section of Anatomy and Histology, University of Florence, Florence, Italy; Albert Einstein College of Medicine, United States of America

## Abstract

The hormone relaxin (RLX) is produced by the heart and has beneficial actions on the cardiovascular system. We previously demonstrated that RLX stimulates mouse neonatal cardiomyocyte growth, suggesting its involvement in endogenous mechanisms of myocardial histogenesis and regeneration. In the present study, we extended the experimentation by evaluating the effects of RLX on primary cultures of neonatal cardiac stromal cells. RLX inhibited TGF-β1-induced fibroblast-myofibroblast transition, as judged by its ability to down-regulate α-smooth muscle actin and type I collagen expression. We also found that the hormone up-regulated metalloprotease (MMP)-2 and MMP-9 expression and downregulated the tissue inhibitor of metalloproteinases (TIMP)-2 in TGF-β1-stimulated cells. Interestingly, the effects of RLX on cardiac fibroblasts involved the activation of Notch-1 pathway. Indeed, Notch-1 expression was significantly decreased in TGF-β1-stimulatedfibroblasts as compared to the unstimulated controls; this reduction was prevented by the addition of RLX to TGF-β1-stimulated cells. Moreover, pharmacological inhibition of endogenous Notch-1 signaling by *N*-3,5-difluorophenyl acetyl-L-alanyl-2-phenylglycine-1,1-dimethylethyl ester (DAPT), a γ-secretase specific inhibitor, as well as the silencing of Notch-1 ligand, Jagged-1, potentiated TGF-β1-induced myofibroblast differentiation and abrogated the inhibitory effects of RLX. Interestingly, RLX and Notch-1 exerted their inhibitory effects by interfering with TGF-β1 signaling, since the addition of RLX to TGF-β1-stimulated cells caused a significant decrease in Smad3 phosphorylation, a typical downstream event of TGF-β1 receptor activation, while the treatment with a prevented this effect. These data suggest that Notch signaling can down-regulate TGF-β1/Smad3-induced fibroblast-myofibroblast transition and that RLX could exert its well known anti-fibrotic action through the up-regulation of this pathway. In conclusion, the results of the present study beside supporting the role of RLX in the field of cardiac fibrosis, provide novel experimental evidence on the molecular mechanisms underlying its effects.

## Introduction

Relaxin (RLX) is a polypeptide hormone with well-recognized antifibrotic properties [1], [2]. Although first described as a reproductive hormone causing an increase in uterine cervix collagen solubility and softening of the pubic symphysis during pregnancy [3], evidence is accumulating that it is also able to regulate extracellular matrix (ECM) remodeling in organs and tissues other than the reproductive system, such as the heart, liver, kidney, and lung [1], [2], [4]–[7]. Indeed, RLX knock-out (KO) mice have been shown to develop age-associated pulmonary, renal and left ventricular fibrosis, which can be reversed by the administration of exogenous RLX [8]–[11]. Moreover, the treatment with RLX or RXFP1 (RLX receptor) agonists induces a collagen-degrading phenotype in a variety of experimental settings, including bleomycin-induced lung and peri-bronchiolar fibrosis [12], [13] and prevents fibrosis associated with skeletal muscle injury [14], [15]. Several *in vitro* and *in vivo* studies have reported that the effects of RLX on collagen deposition are mainly mediated by its ability to inhibit TGF-β1-induced fibroblast-myofibroblast transition [16]–[19] and stimulate the expression and release of ECM-degrading enzymes by a large number of cells. The fact that RLX has no effects on basal collagen expression [19] have sparkled interest in the therapeutic potential of this hormone as an anti-fibrotic agent, so that clinical trials have been carried out to test its effectiveness in the treatment of diseases characterized by a prominent component of fibrosis [20]–[23].

Notch-1 is part of a highly conserved signaling pathway between adjacent cells, which involves the expression of receptors (Notch-1/4) and cognate transmembrane ligands (Jagged-1/2) that mediate key cell fate decisions during development and in differentiated tissues. Upon ligand binding, the Notch intracellular domain (NICD) is released by proteolytic cleavage operated by γ-secretase complex, migrates into the nucleus and interacts with transcriptional repressors [24]. Of interest, Notch signaling has been recently shown to be involved in tissue fibrosis: depending on cells and stimuli, it can positively or negatively influence the switching of fibroblasts into myofibroblasts [25]–[28]. In particular, over-expression of Notch-1 has been shown to facilitate lung fibroblast transition into myofibroblasts [27]. On the other hand, it has been reported that Notch-1 can inhibit the conversion of hepatic stellate cells and 10T1/2 fibroblasts into myofibroblasts [26], that Jagged-1 over-expressing fibroblasts are refractory to the profibrotic effects of TGF-β1- [29], and that inhibition of Notch signaling by a highly active γ-secretase inhibitor N-[N-(3,5-difluorophenacetyl)-l-alanyl]-S-phenylglycine t-butyl ester (DAPT) promotes cardiac fibroblast-myofibroblast transition [25]. Along this line of evidence, it has been recently demonstrated that TGF-β1 limits Notch-1 activation in mouse fibroblasts by inducing the expression of ADAM12 [30], [31], a member of metalloprotease-disintegrin family, which is capable of cleaving Notch ectodomain after ligand-receptor interaction [32], [33]. This finding suggests a functional antagonism between TGF-β1 and Notch-1 signaling.

On the basis of these observations, the aim of this study was to investigate the role of Notch-1 signaling in primary cultures of mouse cardiac stromal cells and NIH/3T3 fibroblasts and to evaluate whether RLX could exert its anti-fibrotic effects through the down-regulation of fibroblast-myofibroblast transition operated by Notch-1 signaling.

## Materials and Methods

### Ethics Statements

Animal handling and use complied with the European Community guidelines for animal care (DL 116/92, application of the European Communities Council Directive of 24 November 1986; 86/609/EEC) and were approved by the Committee for Animal Care and Experimental Use of the University of Florence. The ethical policy of the University of Florence conforms to the Guide for the care and use of laboratory animals of the U.S. National Institutes of Health (NIH Publication No. 85–23, revised 1996; University of Florence assurance No. A5278-01). The protocols were communicated to local authorities and to Italian Ministry of the Health; according to the Italian law (Art.7/D.lgs 116/92) such procedure doesn’t require Ministry authorization. The animals had free access to food and water and were housed on a 12 h light/dark cycle at 22°C room temperature. The experiments were designed to minimize pain and the number of animals used. Sacrifice was carried out by decapitation.

### Cell Culture and Treatments

Murine fibroblastic NIH/3T3 cells, obtained from American Type Culture Collection (ATCC, Manassas, VA, USA), were cultured in Dulbecco’s modified Eagle’s medium (DMEM) supplemented with 10% fetal bovine serum (FBS), penicillin (100 U/ml) and streptomycin (100 U/ml) (Sigma, Milan, Italy) at 37°C in a humidified atmosphere of 5% CO_2_.

Primary cultures of mouse cardiac stromal cells were prepared from hearts of 1-day old newborn CD1 albino mice (Harlan, Correzzana, Italy) according to Formigli *et al.* [34], with modifications. Briefly, hearts were quickly excised, the atria were cut-off and the ventricles minced and digested at 37°C for 45 min in calcium-free HEPES-buffered Hanks’ solution, pH 7.4, containing 100 g/ml type II collagenase (Invitrogen, Carlsbad, CA, USA). The tissue lysate was filtered through a 70 µm cell strainer (Millipore, Billerica, MA, USA) and pre-plated for 1 h. Cardiomyocyte-rich cell suspension was discarded while the cells adherent to the culture plate, mainly composed by stromal cells, were cultured in DMEM supplemented with 20% FBS, penicillin (100 U/ml) and streptomycin (100 U/ml) (Sigma) at 37°C in a humidified atmosphere of 5% CO_2_. When the cultures reached about 80% confluence, the adherent cells were detached with 0.05% trypsin-0.03% EDTA (Invitrogen) for 5 min at 37°C, washed with PBS, re-suspended in DMEM and plated on tissue culture dishes (P1) to be used for the experiments. Aliquots of cells at P1 culture passage were seeded on glass coverslips and assayed for vimentin immunophenotype by confocal microscopy to confirm their stromal nature and to test the degree of purity of the cell cultures, which usually was 92–98%.

Both NIH/3T3 fibroblasts and primary cardiac stromal cells (P1) were induced to transform into myofibroblasts in DMEM containing 2% FBS for different times (24 h, 48 h, 72 h, 5 days) in the absence (control) or presence of 2 ng/ml human TGF-β1 (PeproTech, Inc., Rocky Hill, NJ, USA). In some experiments human recombinant H2 RLX (kindly provided by Prof. Mario Bigazzi, Foundation for the Study of Relaxin in Cardiovascular and Other Diseases, Prosperius Institute, Florence, Italy) was added to the culture medium at a 100 ng/ml final concentration. This RLX concentration was similar to that used previously to induce growth and differentiation of mouse neonatal cardiomyocytes [35]. Some experiments were performed treating the cells with the γ-secretase inhibitor, *N*-3,5-difluorophenyl acetyl-L-alanyl-2-phenylglycine-1,1-dimethylethyl ester (DAPT; 5 µM, stock 5 mM in dymethil sulfoxide, DMSO, 0.1%, Sigma), which blocks the generation of NICD and hence Notch-1 signaling.

### Total RNA Extraction and Reverse Transcription (RT)-PCR

Expression levels of mRNA for RLX family peptide receptor 1 (RXFP1) and Notch-1 were assayed by RT-PCR. Total RNA was isolated by extraction with TRIzol Reagent (Invitrogen), according to the manufacturer instructions. One µg of total RNA was reverse transcribed and amplified with SuperScript One-Step RT-PCR System (Invitrogen). After cDNA synthesis for 30 min at 55°C, the samples were pre-denatured for 2 min at 94°C and then subjected to 40 cycles of PCR performed at 94°C for 15 s, alternating with 55°C for RXFP1, 56°C for Notch-1, and 57°C for GAPDH for 30 s and 72°C for 1 min; the final extension step was performed at 72°C for 5 min. The following mouse gene-specific primers were used: RXFP1 (NM_ 212452.1), forward 5′-ACG AGC TGT CCC ATC AGT TT-3′and reverse 5′-ATG TGC TGA CAG AGG GGT TT-3′; Notch-1 (NM_008714.3), forward 5′-GTC CCA CCC ATG ACC ACT AC-3′ and reverse 5′-CCT GAA GCA CTG GAA AGG AC-3′ and GAPDH (NM_ 008084.2), forward 5′-CGTCCCGTAGACAAAATGGT-3′ and reverse 5′-TCAATGAAGGGGTCGTTGAT -3′. GAPDH mRNA was used as internal standard. Blank controls, consisting in no template (water), were performed in each run. PCR products were electrophoresed on a 2% agarose gel and the ethidium bromide-stained bands were quantified by densitometric analysis using Scion Image Beta 4.0.2 image analysis program (Scion Corp., Frederick, MD, USA). GAPDH normalization was performed for each result.

### Western Blotting

Cells were resuspended in appropriate volume of ice-cold cell extraction buffer (10 mM Tris/HCl, pH 7.4, 100 mM NaCl, 1 mM EDTA, 1 mM EGTA, 1 mM NaF, 20 mM Na_4_P_2_O_7_, 2 mM Na_3_VO_4_, 1% Triton X-100, 10% glycerol, 0.1% SDS, 0.5% deoxycholate; Invitrogen) supplemented with 50 µl/ml protease inhibitor cocktail and 1 mM phenylmethanesulfonyl fluoride, PMSF (Sigma). Upon centrifugation at 13,000 g for 10 min at 4°C, the supernatants were collected and the total protein content was quantified using Qubit Protein assay Kit (Molecular Probes, Eugene, OR) following the manufacturer instructions. Forty µg of total proteins were electrophoresed on NuPAGE® 4–12% Bis-Tris Gel (Invitrogen; 200 V, 40 min) and blotted onto polyvinylidene difluoride (PVDF) membranes (Invitrogen; 30 V, 1 h). The membranes were blocked with blocking solution included in the Western Breeze® Chromogenic Western Blot Immunodetection Kit (Invitrogen) for 30 min at room temperature on rotary shaker and incubated overnight at 4°C with rabbit polyclonal anti relaxin receptor (RXFP; 1∶3000, Immunodiagnostik, Bensheim, Germany), mouse monoclonal anti -α smooth muscle actin (sma, 1∶1000, Abcam, Cambridge, UK), rabbit polyclonal anti- type I collagen (1∶50; Santa Cruz Biotechnology, Santa Cruz, CA, USA), rabbit polyclonal anti-metalloprotease (MMP)-2 (1∶2000, Abcam), rabbit polyclonal anti-MMP-9 (1∶1000, Abcam), rabbit monoclonal anti-Notch-1 (1∶2000; Abcam), goat polyclonal anti-Jagged-1 (1∶1000; Santa Cruz Biotechnology), rabbit monoclonal anti-Smad3 (1∶1000; Cell Signaling Technology, Danvers, MA, USA) and rabbit polyclonal anti-glyceraldehyde 3-phosphate dehydrogenase (GAPDH, 1∶1000, Cell Signaling Technology) antibodies, assuming GAPDH as control invariant protein. Immunodetection was performed as described in the Western Breeze®Chromogenic Immunodetection protocol (Invitrogen). Densitometric analysis of the bands was performed using ImageJ software (http://rsbweb.nih.gov/ij) and the values normalized to GAPDH.

### Confocal Immunofluorescence

Cells grown on glass coverslips were fixed with 0.5% buffered paraformaldehyde (PFA) for 10 min at room temperature. After permeabilization with cold acetone for 3 min, the fixed cells were blocked with 0.5% bovine serum albumin (BSA; Sigma) and 3% glycerol in PBS for 20 min and then incubated overnight at 4°C with the following primary antibodies: mouse monoclonal anti-vimentin (1∶100, Santa Cruz); mouse monoclonal anti-vinculin (1∶100; Sigma); mouse monoclonal anti-α-sma (1∶100, Abcam), rabbit polyclonal anti- type I collagen (1∶50, Santa Cruz); rabbit polyclonal anti-MMP-2 (1∶200; Abcam), rabbit polyclonal anti-MMP-9 (1∶100, Abcam), rabbit monoclonal anti-Notch-1 (1∶200, Abcam), mouse monoclonal anti-tissue inhibitor of metalloproteinase-2 (TIMP-2, 1∶20, Abcam), and rabbit polyclonal anti-Hes-1, a typical Notch-1 transcriptional target gene (1∶200, Millipore) antibodies. Immunoreactions were revealed by specific anti-rabbit or anti-mouse Alexa Fluor 488-conjugated IgG (1∶200; Molecular Probes) for 1 h at RT. In some experiments, counterstaining was performed with either TRITC-labeled phalloidin (1∶100; Sigma) to reveal stress fibers, propidium iodide (1∶30 for 30 s at RT; Molecular Probes) or Syto16 (1∶1000 for 3 min at RT; Molecular Probes) to reveal nuclei. Negative controls were carried out by replacing the primary antibodies with non-immune mouse or rabbit serum, as appropriate. Negative control experiments were performed by omitting the primary antibodies. After washing, the immunolabeled cells were mounted in antifade medium (Biomeda Gel Mount, Electron Microscopy Sciences, Foster City, CA, USA) and observed under a confocal Leica TCS SP5 microscope (Leica Microsystems, Mannheim, Germany) equipped with a HeNe/Ar laser source for fluorescence measurements. Observations were performed using a Leica Plan Apo 63X/1.43NA oil immersion objective. Series of optical sections (1024×1024 pixels each; pixel size 204.3 nm) 0.4 µm in thickness were taken through the depth of the cells at intervals of 0.4 µm. Images were then projected onto a single ‘extended focus’ image. When needed, a single optical fluorescent section and DIC images were merged to view the exact distribution of the immunostaining. Densitometric analyses of the intensity of F-actin, vinculin, α-sma, MMP-2, MMP-9, TIMP-2, Notch-ICD, and nuclear Hes-1 fluorescent signals were performed on digitized images using ImageJ software (http://rsbweb.nih.gov/ij) in 20 regions of interest (ROI) of 100 µm^2^ for each confocal stacks (at least 10).

#### Silencing of jagged-1 by siRNA

To inhibit the expression of Jagged-1, short interference RNA duplexes (siRNA) (Santa Cruz) were used essentially as describred previously [36]. A non-specific scrambled (SCR) siRNA (Santa Cruz) was used as control. Primary neonatal cardiac fibroblasts (P1) were transfected using siRNA trasfection medium (Santa Cruz) with the mixed combination of Jagged-1 siRNA duplexes or with SCR-siRNA (20 nM) in serum and antibiotic free medium. After 5 h, transfected cells were cultured in fresh medium containing FBS for additional 24 h (T0), and then shifted in DMEM with 2% FBS and penicillin/streptomycin, stimulated or not with TGF-β1 (2 ng/ml) in the presence or absence of RLX, for 24 h. Specific knock-down of Jagged-1 was evaluated by confocal immunofluorescence (not shown) and Western blotting. The efficiency of transfection was estimated to be approximately 70%.

### Immunoprecipitation

The cells were lysed as reported previously [37]. One mg of whole cell extract was pre-cleared by Protein A/G Plus-Agarose (Santa Cruz Biotechnology) for 1 h at 4°C. After centrifugation, the supernatants were collected in tubes and incubated overnight at 4°C with monoclonal rabbit anti-Smad3 antibody diluted 1∶100 (Cell Signaling Technology), gently inverting the tubes. Then the samples were re-incubated with Protein A/G Plus-Agarose for 2 h at 4°C and precipitated by centrifugation. Complexes were subjected to electrophoresis and blotted with mouse monoclonal anti-phospho-tyrosine antibody (1∶1000; Santa Cruz Biotechnology) and then re-probed with rabbit monoclonal anti-Smad3 antibody (1∶1000; Cell Signaling Technology).

### Statistical Analysis

Data were reported as mean ± SEM. Unless otherwise stated, statistical significance was determined by one-way ANOVA and Newman-Keuls multiple comparison test or Student’s *t* test. A *p* value≤0.05 was considered significant. Calculations were performed using GraphPad Prism software (GraphPad, San Diego, CA, USA).

## Results

### Relaxin Inhibits TGF-β-stimulated Cardiac Fibroblast-myofibroblast Transition

**Figure 1:**
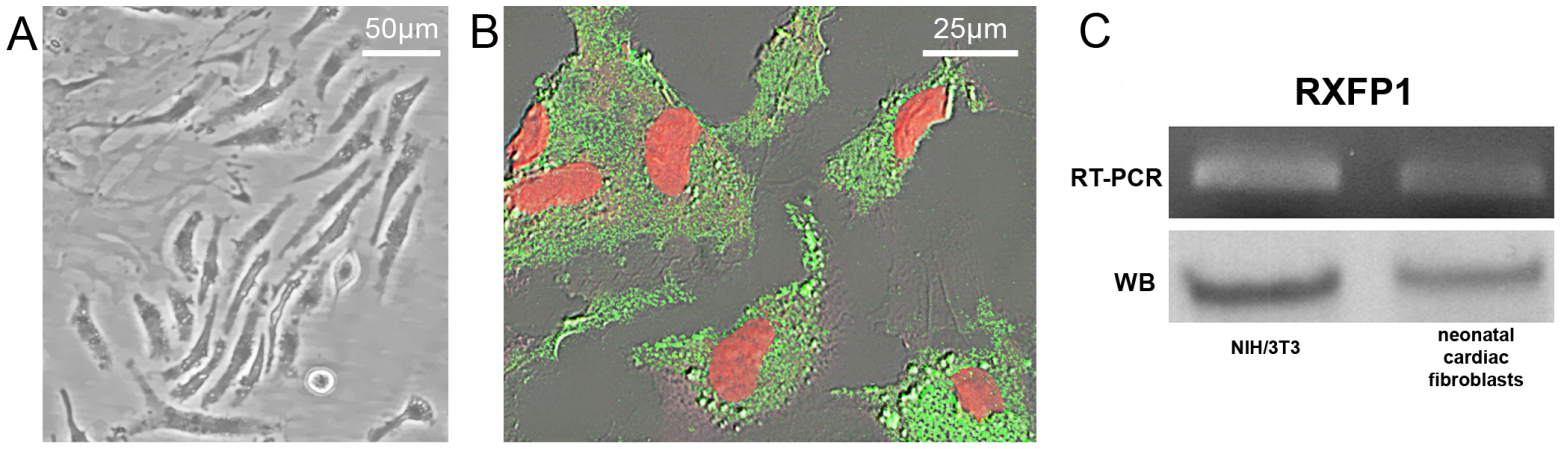
Characterization and expression of RLX receptor on primary neonatal cardiac fibroblasts. A) Representative contrast phase microscopy images of first passage neonatal murine neonatal cardiac fibroblasts. B) Representative superimposed differential interference contrast (DIC) and confocal immunofluorescence images of neonatal cardiac fibroblasts immunostained with antibodies against vimentin (green). Nuclei are counterstained in red with propidium iodide. C) Expression of Relaxin family peptide receptor 1 (RXFP1) in neonatal cardiac fibroblasts and NIH/3T3 at mRNA level determined by RT-PCR, and protein level evaluated by Western blotting analysis. The images are representative of at least three independent experiments with similar results.

**Figure 2:**
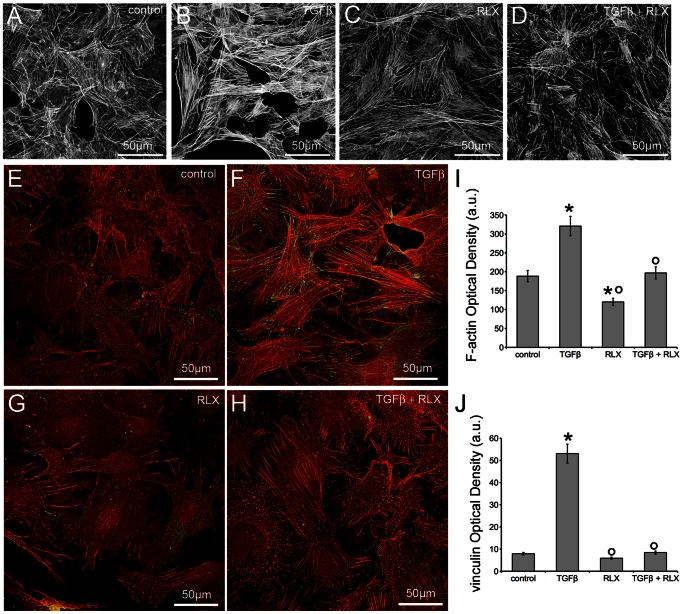
Relaxin attenuates TGF-β1 induced cytoskeletal assembly in NIH/3T3 and primary neonatal cardiac fibroblasts. Representative confocal immunofluorescence images of (A–D) NIH/3T3 cells stained with TRITC-phalloidin to reveal F-actin and of (E–H) primary cardiac fibroblasts stained with TRITC-phalloidin (red) and anti-vinculin antibody (green) to detect focal adhesions, cultured for 48 h in the indicated experimental conditions. I, J) Densitometric analyses of the intensity of F-actin and vinculin fluorescence signals performed on digitized images of neonatal cardiac fibroblasts. The images are representative of at least three independent experiments with similar results. Significance of differences: *p<0.05 *vs* control, °p<0.05 *vs* TGF-β.

**Figure 3:**
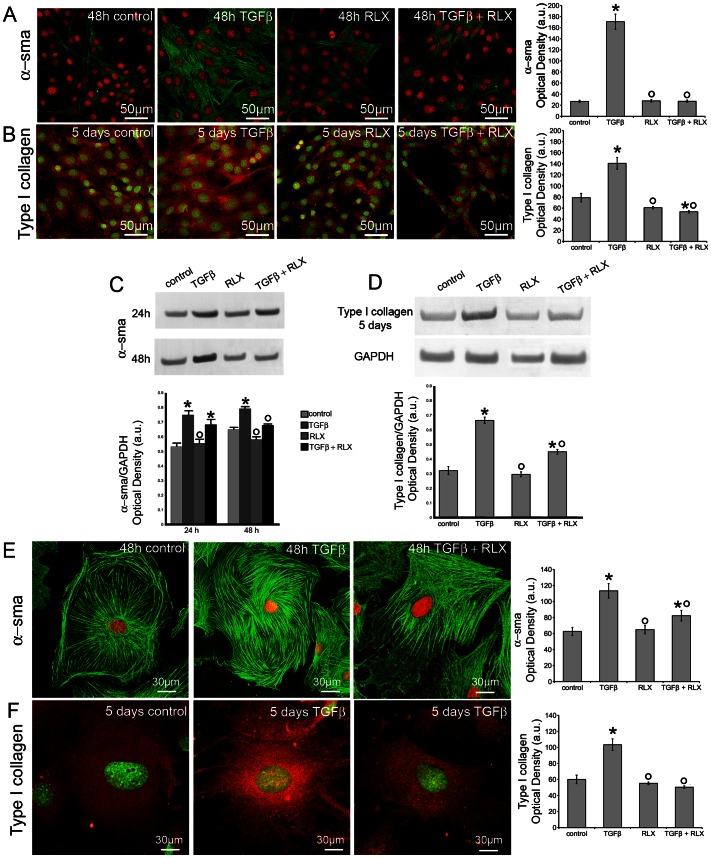
Relaxin reduces α–sma and type I collagen expression in TGF-β1 treated NIH/3T3 and primary neonatal cardiac fibroblasts. A,B, E,F) Representative confocal immunofluorescence images of NIH/3T3 cells (A,B) and primary cardiac fibroblasts (E, F) cultured in the indicated experimental conditions and immunostained with antibodies against α–sma (A,E; green) or type I collagen (B, F, red). Nuclei are labeled (A,E) with propidium iodide (red) or with (B,F) Syto16 (green). The histograms show the corresponding densitometric analyses of the intensity of α–sma and type I collagen fluorescence signals. C,D) Western blotting analyses of the expression of α–sma and type I collagen proteins in neonatal cardiac fibroblasts. The densitometric analysis of the bands normalized to GAPDH is reported in the histograms. Data are representative of at least three independent experiments with similar results. Significance of differences: *p<0.05 *vs* control, °p<0.05 *vs* TGF-β1.

**Figure 4:**
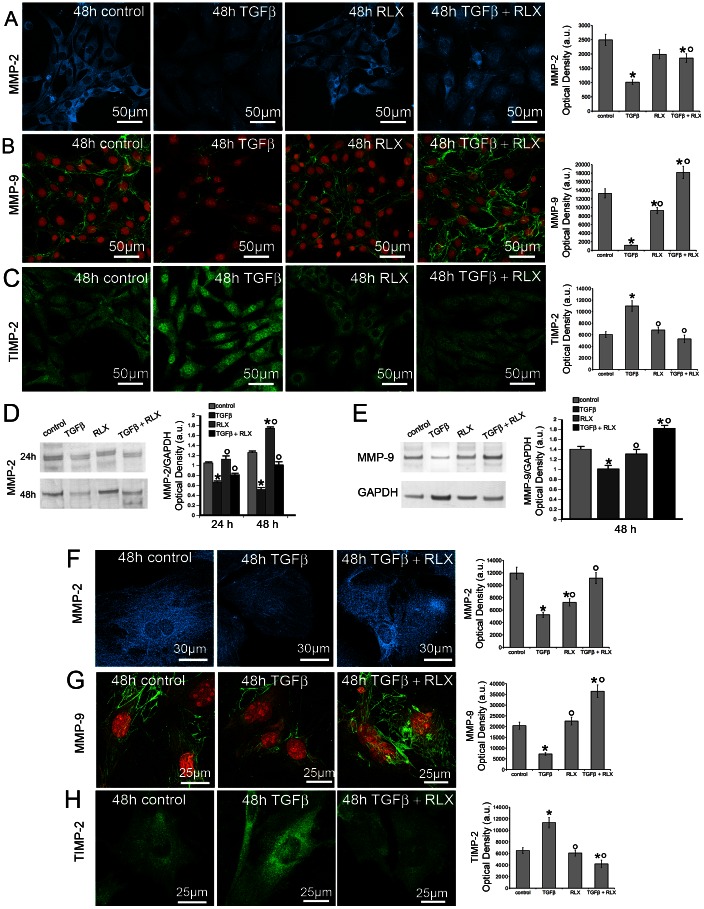
Relaxin prevents TGF-β1 induced down regulation of MMP-2 and MMP-9 expression and up-regulation of TIMP-2 in NIH/3T3 and primary neonatal cardiac fibroblasts. A–C, F–H). Representative confocal immunofluorescence images of NIH/3T3 cells (A–C) and primary neonatal cardiac fibroblasts (F–H) cultured in the indicated experimental conditions and immunostained with antibodies against MMP-2 (A,F; cyan), MMP-9 (B,G; green) or TIMP-2 (C,H, green). In B and G the nuclei are labeled in red with propidium iodide. The histograms show the corresponding densitometric analyses of the intensity of MMP-2, MMP-9 and TIMP-2 fluorescence signals. D–E) Western blotting analyses of the expression of (D) MMP-2, and (E) MMP-9 proteins in neonatal cardiac fibroblasts. In the histograms the densitometric analyses of the bands normalized to GAPDH are reported. Data are representative of at least three independent experiments with similar results. Significance of differences: *p<0.05 *vs* control, °p<0.05 *vs* TGF-β1.

Neonatal cardiac stromal cells at P1 culture passage were first characterized morphologically and immunophenotypically: they showed the typical features of fibroblasts with elongated polygonal or spindle shape, large ovoid nuclei (Fig. 1A) and strong vimentin immunoreactivity (Fig. 1B). Then, the expression of RXFP by NIH/3T3 fibroblasts and neonatal cardiac stromal cells was ascertained by RT-PCR and Western blotting (Fig. 1C). On these cells, we evaluated the effects of RLX on fibroblast-myofibroblast transition. To this aim, NIH/3T3 fibroblasts and neonatal cardiac stromal cells were grown in low serum medium with a added with TGF-β1 (2 ng/ml), a well-known pro-fibrotic factor, in the absence or presence of RLX (100 ng/ml) for 48 h. Confocal immunofluorescence analysis showed that TGF-β1 induced a prominent cytoskeletal rearrangement, consisting in the formation of robust stress fibres and focal adhesion sites (Fig. 2). As judged by confocal immunofluorescence and Western blotting analyses, this effect was associated with an increase in both α-sma, a marker of myofibroblasts, and type I collagen expression (Fig. 3). In particular, α-sma was mainly localized along the stress fibres (Fig. 3A,E), whereas type I collagen was mainly distributed throughout the cytoplasm in both NIH/3T3 and cardiac fibroblasts (Fig. 3 B,F). We also found that the levels of MMP-2 and MMP-9 decreased in TGF-β1-stimulated cells (Fig. 4A,B, D–G); these enzymes showed distinct cellular distributions, being MMP-2 mainly located inside the cytoplasm (Fig. 4 A,F) and MMP-9 concentrated along cytoskeletal filaments (Fig. 4 B,G). Moreover, the expression of TIMP-2 was significantly increased in the stimulated NIH/3T3 cells (Fig. 4 C) and cardiac fibroblasts (Fig. 4H), in agreement with data showing that high TIMP-2 levels have a potent inhibitory action on MMP activation [38].

RLX alone did not affect the morphological pattern of the unstimulated NIH/3T3 fibroblasts and cardiac stromal cells, whose cytoskeletal apparatus, as well as the expression of α-sma, type I collagen, MMP-2, MMP-9 and TIMP-2, appeared unmodified by RLX addition as compared with the unstimulated control cells (Figs. 2–4). On the other hand, RLX was able to strongly reduce the phenotypical changes induced by TGF-β1, based on the findings that it was able to: *i)* inhibit stress fiber assembly and re-distribution of vinculin to focal adhesion sites (Fig. 2); *ii)* prevent the up-regulation of α-sma (Fig. 3A,C, E) and type I collagen (Fig. 3 B,D,F); *iii)* reduce the down regulation of MMP-2 and MMP-9 (Fig. 4 A,B, D–G) and, *iv)* reduce the expression of TIMP-2 (Fig. 4C,H), in TGF-β1-stimulated cells.

### Notch-1 Pathway is Involved in RLX-mediated Inhibition of Cardiac Fibroblast-myofibroblast Transition

**Figure 5:**
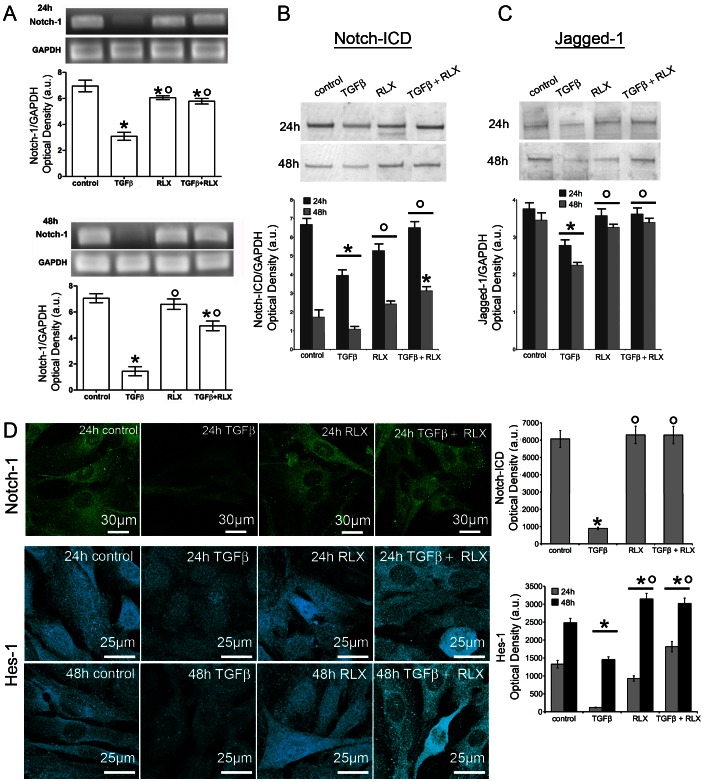
Relaxin prevents the TGF-β1-induced down-regulation of Notch-1 pathway in primary neonatal cardiac fibroblasts. A) RT-PCR of Notch-1 expression. B,C) Western blotting analysis of activated intracellular form of Notch-1 (Notch-ICD, B) ) and of Notch-1 ligand, Jagged-1(C). The densitometric analyses of the bands normalized to GAPDH are reported in the histograms. D) Confocal immunofluorescence analysis of Notch-1 (green) and Hes-1 (cyan) expression. For the analysis of Notch-1, the cells were stained with a specific antibody recognizing both the membrane Notch-1 receptor and its activated intracellular form, Notch-ICD. Densitometric analyses of Notch-ICD and Hes-1 fluorescent signals are reported in the corresponding histogram. Significance of differences: *p<0.05 *vs* control, °p<0.05 *vs* TGF-β1.

**Figure 6:**
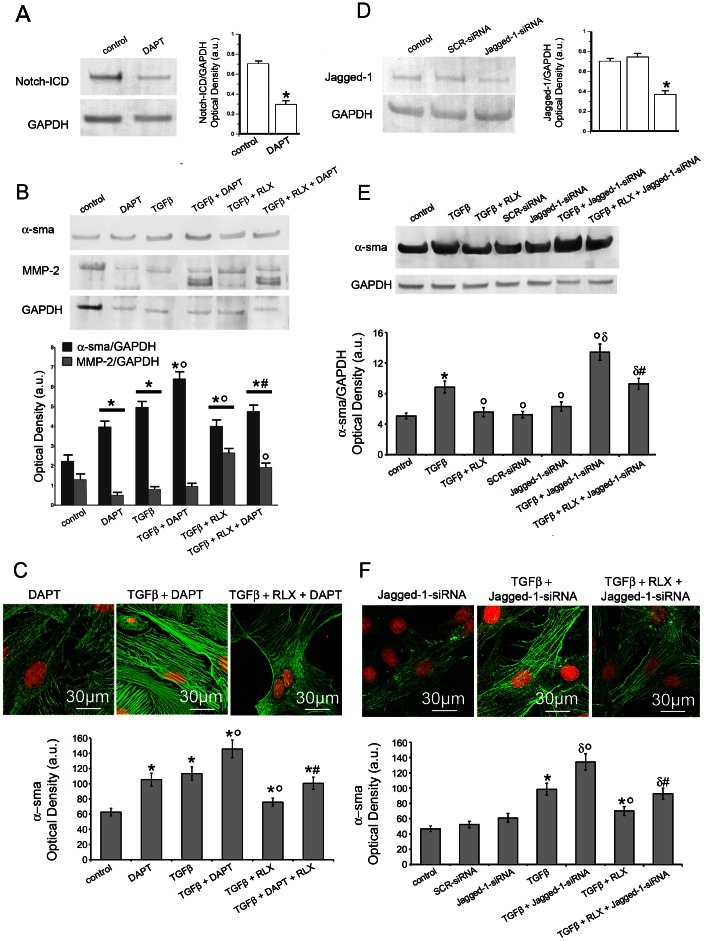
RLX and Notch-1 negatively regulates TGF-β1-induced fibroblast-myofibroblast transition in cardiac fibroblasts. Neonatal cardiac fibroblasts were cultured for 48 h and treated as indicated. A) Western blotting analysis of NICD expression in the absence (control) or presence of DAPT (5 µM) a pharmacological γ-secretase inhibitor, used to block the generation of NICD. B) Western Blotting analysis of α–sma and MMP-2 expression in the cells treated with DAPT. C) Representative confocal immunofluorescence images cardiac fibroblasts treated with DAPT, fixed and stained with antibodies against α–sma (green). Nuclei are marked in red with propidium iodide. D) Western blotting analysis of Jagged-1 expression in control cells, cells transfected with non specific scrambled-siRNA (SCR-siRNA) or silenced for the expression of Notch-1 ligand, Jagged-1, by specific Jagged-1 siRNA (Jagged-1 siRNA). E) Western Blotting analysis of α–sma in Jagged-1 silenced cells. F) Representative confocal immunofluorescence images cardiac fibroblasts silenced for Jagged-1 expression, fixed and stained with antibodies against α–sma (green). Nuclei are marked in red with propidium iodide. The densitometric analyses of the bands normalized to GAPDH are reported in histograms in A–E; the densitometric analyses α–sma fluorescent signal are shown in the histograms in and C, F. Significance of differences: *p<0.05 *vs* control, ^δ^p<0.05 vs SCR-siRNA, °p<0.05 *vs* TGF-β1, ^#^p<0.05 vs TGF-β1+ RLX.

To investigate the molecular mechanisms underlying the inhibitory effect of RLX on TGF-β1-induced fibroblast-myofibroblast transition, we next investigated the involvement of Notch-1 signaling, which has been implicated in the process of tissue fibrosis [26]–[28]. By RT-PCR, Notch-1 mRNA expression was detected in NIH/3T3 cells (data not shown) and neonatal cardiac stromal cells after 24 h and 48 h of culture (Fig. 5A); this expression was dramatically reduced by the administration of TGF-β1 as compared with the unstimulated control cells (Fig. 5A). As demonstrated by Western blotting, TGF-β1 also caused a significant decrease of the levels of NICD, the intracellular form of activated Notch-1 as well as of Notch-1 ligand, Jagged-1 (Fig. 5B, C). By confocal immunofluorescence, using a specific antiserum recognizing both Notch-1 receptor and NICD, we confirmed the reduced expression of both Notch-1 receptor at the plasma membrane and NICD within the cytoplasm and nucleus in the TGF-β1-stimulated cells as compared with the unstimulated controls (Fig. 5D). Moreover, in keeping with the reduced expression and activation of Notch-1, the expression of Hes-1, a canonical Notch-1 transcriptional target gene, was also significantly down-regulated by TGF-β1 (Fig. 5D), confirming the notion of a functional antagonisms between TGF-β1 and Notch-1 in cardiac fibroblasts. The administration of RLX to unstimulated fibroblasts did not affect the basal expression of Notch-1 and Jagged-1, and induced a slight increase in the levels of Hes-1 (Fig. 5 A–D). Interestingly, the hormone prevented the reduction of Notch-1, Jagged-1 and Hes-1 induced by TGF-β1 (Fig. 5 A–D). Furthermore, the pharmacological inhibition of endogenous Notch-1 signaling by DAPT (5 µM) which blocks the generation of NICD (Fig. 6A), as well as the silencing of Notch-1 ligand, Jagged-1 (Fig. 6D), were both capable of potentiating TGF-β1-induced generation of myofibroblasts, as judged by increased α–sma (Fig. 6 B,C,E,F) and reduced MMP-2 (Fig. 6B) in the treated cardiac fibroblasts. As expected, the treatment with DAPT and Jagged-1-siRNA also abrogated the inhibitory effects of RLX on TGF-β1-induced fibroblast-myofibroblast transition (Fig. 6 B,C,E,F).

**Figure 7:**
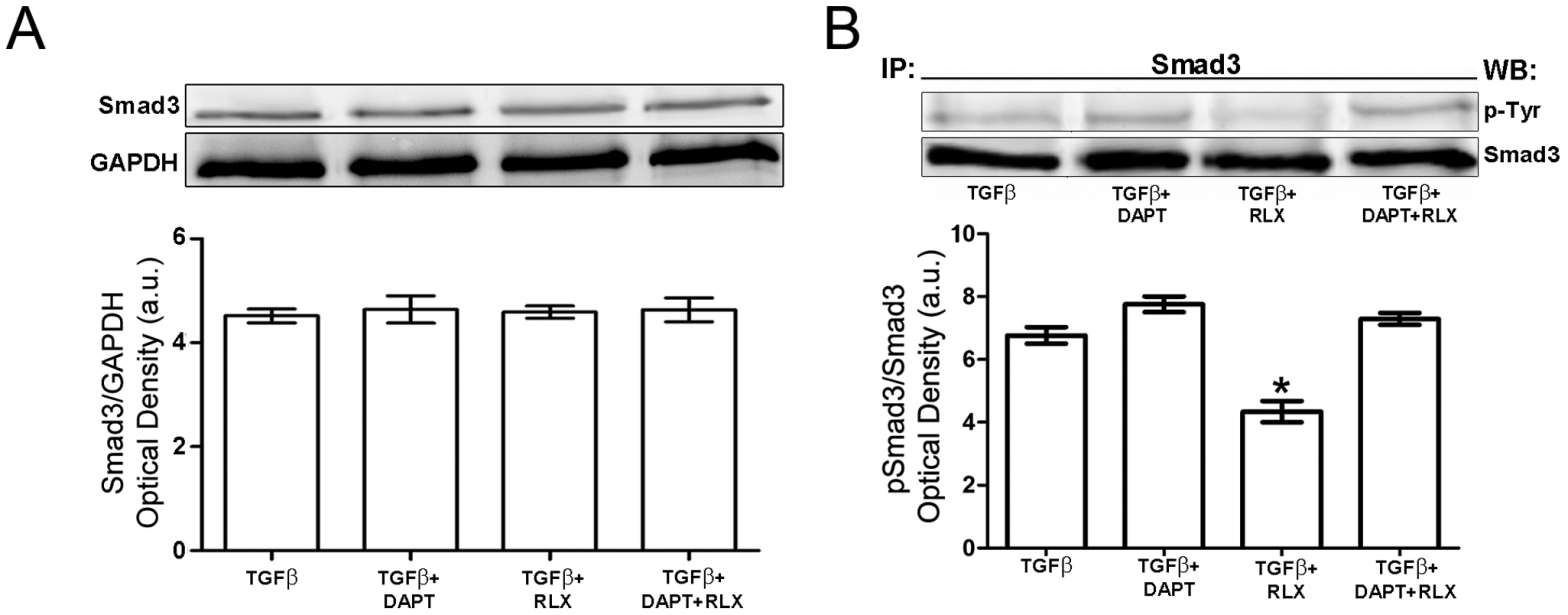
RLX and Notch-1 negatively regulates TGF-β1 signaling in primary neonatal cardiac fibroblasts. (A) Western blotting analysis of Smad3 expression and (B) phosphorylation (performed on the immunoprecipitated Smad3 proteins) in cardiac fibroblasts cultured for 48 h in the indicated experimental conditions. Data are representative of at least three independent experiments. Histograms show the densitometric analyses of the bands normalized to (A) GAPDH and (B) total Smad3. Significance of differences: *p<0.05 *vs* TGF-β1.

Finally, we demonstrated that RLX and Notch-1 exerted their antifibrotic effects interfering with the TGF-β1-mediated intracellular signaling. In fact, the addition of RLX to TGF-β1-treated cardiac fibroblasts caused a marked decrease in phosphorylated Smad3 (pSmad3), the TGF-β1 downstream signaling molecule, without modifying its expression (Fig. 7). Conversely, the addition of DAPT to TGF-β1-stimulated cells increased the levels of pSmad3 and prevented the inhibitory effects of RLX (Fig. 7).

Taken together, these findings suggest that Notch-1 signaling can negatively regulate TGF-β1/Smad3-mediated fibroblast-myofibroblast transition and that RLX could exert its anti-fibrotic action through the up-regulation of this pathway.

## Discussion

The balance between collagen synthesis and degradation after tissue injury, as occurs upon myocardial infarction, is regulated by myofibroblasts. These cells mainly originate from cardiac fibroblasts [39], [40], andrespond to mechanical stretch, ischemia, autocrine and paracrine factors - such as angiotensin II, TGF-β1 and pro-inflammatory cytokines - by increasing synthesis and deposition of ECM proteins which replace the necrotic myocardium with a scar. The most typical feature of these cells is *de novo* formation and deployment of stress fibers, the expression of smooth muscle genes, such as α-sma and collagen synthesis and deposition. Although myofibroblasts are required to stabilize the infarcted area and promote scar tissue contraction, their persistence can contribute to abnormal myocardial stiffness and impairment of ventricular function. Along this line, identification of the factors and mechanisms capable of preventing myofibroblast generation and defining their molecular targets appears a key step for the design of therapeutic strategies aimed at inhibiting excessive left ventricular stiffness after myocardial infarction. In the present study, we have demonstrated that RLX can inhibit cardiac fibroblast-myofibroblast transition *in vitro* by interfering with TGF-β1 signaling. This new achievement confirms and extends the knowledge on the anti-fibrotic action of this hormone and on its beneficial actions in the cardiovascular system under pathological conditions [1], [2], [4]–[6]. In particular, our results have shown that RLX, administered to TGF-β1-stimulated NIH/3T3 fibroblasts and cardiac stromal cells, down-regulates multiple processes involved in adverse cardiac remodeling, causing inhibition of stress fibers and formation of focal adhesion sites which play a key role in enhancing the promoter activity of collagen genes [41], reduction of α-sma and type I collagen expression, and up-regulation of MMP-2 and MMP-9. In turn, MMPs can degrade collagen and other ECM proteins and contribute to tissue remodeling. It is well accepted that ECM homeostasis is also dependent on a fine coordination between these proteolytic enzymes and their specific tissue inhibitors (TIMPs) [42], [43]. In a such a view our data showing the ability of RLX to up-regulate MMPs and concomitantly reduce the expression of TIMP-2 in TGF-β1-stimulated cardiac fibroblasts further suggest the central role played by this hormone in promoting ECM turnover and clearance [1], [2].

These effects were likely mediated through the interaction of RLX with its specific receptor RXFP1, which is fairly expressed by both NIH/3T3 fibroblasts and cardiac stromal cells and is known to play a major role in the modulation of RLX-induced tissue remodeling [16], [44], [45].

TGF-β1 signals through trans-membrane receptors, which activate Smad2/3 phosphorylation: in turn, pSmad2/3 complex translocates into the nucleus where they induce the expression of pro-fibrotic target genes [46], [47]. Several distinct mechanisms have been postulated or demonstrated to be involved in RLX-mediated inhibition of TGF-β1/Smad axis including the PI3K-Akt and NOS-NO-cGMP cascades [18], [44], [48]. The results of the present study expand the list of the possible mechanisms underlying the anti-fibrotic action of RLX, demonstrating for the first time that this hormone also acts through Notch-1 up-regulation and activation to inhibit myofibroblast generation and up-regulate MMP expression and activity in NIH/3T3 fibroblasts and cardiac stromal cells. Indeed, we observed that RLX prevented the reduction of Notch-1 expression induced by TGF-β1, and that the pharmacological inhibition of endogenous Notch-1 signaling by DAPT blocked the effects of RLX on inhibition of the fibroblast-myofibroblast transition, the reduction of MMP levels and the down-regulation of pSmad3 in response to TGF-β1 stimulation. The importance of Notch-1 in cardiac myofibroblast generation is consistent with previous *in vitro* observations showing that down-regulation of this pathway in neonatal rat cardiac fibroblasts is necessary for their differentiation into α-sma-positive myofibroblasts [25] and with a recent study on an *in vivo* model of mouse cardiac hypertrophy underscoring its potential significance in cardiac fibrotic response. In particular, it has been demonstrated that Notch-1 controls the balance between fibrotic and regenerative repair in the adult heart, inhibiting myofibroblast generation and promoting mobilization and expansion of cardiac precursors [49].

Previous reports indicate that Notch-1 can interfere with TGF-β1 signaling through a direct interaction of NICD and Smad3 [50]–[52]. This cross-talk may lead to transcriptional cooperation or antagonism, mostly depending on the cell context [46], [53]. Along this line, our observation that Notch-1 is required for RLX-mediated inhibition of the phenotypic switch of cardiac fibroblasts to myofibroblasts offers novel clues to the complex molecular circuitry, which mediates the effects of RLX on ECM turnover. It is conceivable that Notch-1 may cross-talk with other major pathways to inhibit TGF-β1-mediated cardiac fibrosis. Consistently, the recent findings that iNOS, a major signaling molecules of RLX action [54], induces Notch-1 expression and facilitates its signaling [55], imply a mechanistic relationship between these two proteins. Further studies assessing how iNOS and Notch-1 pathways are interlinked and cooperate to mediate the effects of RLX on cardiac fibroblasts will be required to further clarify the mechanisms of the anti-fibrotic action of this hormone and better define its therapeutic range in fibrotic diseases.

In conclusion, the present study offers new evidence that RLX, *via* the up-regulation of Notch-1 signaling, inhibits TGF-β1/pSmad axis and antagonizes the effects of TGF-β1 on cardiac fibroblast-myofibroblast transition. These findings may provide promising prospects for the causative treatment of patients suffering for myocardial fibrosis using RLX or RXFP1agonists.
